# Formation of a Medical Society of South Central New-York

**Published:** 1847-11

**Authors:** 


*Formation of a Medical Society of South Central New-York.*—In our last we published a call for a convention of the Profession of the Counties of Cortland, Tompkins, Tioga and Broome, for the purpose of oranizing a society for said counties. The convention assembled at Owego on the 14th ult., and determined to organize, including, with the aforementioned counties, the county of Chemung. The convention adopted the code of Medical Ethics recommended by the National Convention. The following officers were appointed:—

*President*, Dr. J. S. Paige, of Tioga.
*Vice Presidents*, Dr. G. N. Bredford, of Cortland;
Dr. J. E. Hawley, of Tompkins.
*Recording Secretary*, Dr. H. N. Eastman, of Tioga.
*Assistant Secretary*, Dr. R. Wilcox, of Chemung.
*Corresponding Secretary*, Dr. F. Hyde, of Cortland.
*Treasurer*, Dr. E. Powell, of Tioga.

The Association adjourned to meet at Ithaca, on the first Tuesday of June, 1848. Success to the enterprise!

